# Mechanism of Oleogel‐S10: A triterpene preparation for the treatment of epidermolysis bullosa

**DOI:** 10.1111/dth.12983

**Published:** 2019-07-02

**Authors:** Agnes Schwieger‐Briel, Hagen Ott, Dimitra Kiritsi, Melanie Laszczyk‐Lauer, Christine Bodemer

**Affiliations:** ^1^ Department of Pediatric Dermatology University Childrens' Hospital Zurich Zurich Switzerland; ^2^ Division of Pediatric Dermatology and Allergology Children's Hospital Auf der Bult Hannover Germany; ^3^ Department of Dermatology, Faculty of Medicine Medical Center ‐ University of Freiburg Freiburg Germany; ^4^ Amryt AG Niefern‐Öschelbronn Germany; ^5^ Department of Dermatology Institut Imagine, Necker Enfants Malades Hospital, Paris University, APHP 5; Centre for Genodermatoses (MAGEC); European Network for Rare Skin Diseases (ERN‐SKIN) Paris France

**Keywords:** epidermolysis bullosa, oleogel‐S10, birch bark

## Abstract

Epidermolysis bullosa (EB) is a group of rare heterogeneous, genetic disorders. Currently, there is no effective pharmacological or genetic therapy for all EB subtypes. Dry extract from birch bark and betulin upregulate some pro‐inflammatory mediators and downregulate others. The increase in pro‐inflammatory cytokines is temporary and attenuated over long‐term treatment. This inflammatory stimulus is thought to be prerequisite for a secondary anti‐inflammatory response. Dry extract from birch bark and its active marker substances have also been shown to increase the migration of primary human keratinocytes, accelerate wound closure, and promote differentiation of keratinocytes in vitro and in vivo—processes that are essential for reepithelialization and maintenance of the skin barrier. Comprehensive clinical data are available to support the use of Oleogel‐S10 in the treatment of partial thickness wounds of different etiologies, and a proof‐of‐concept Phase 2 study in patients with dystrophic EB has suggested the potential for faster reepithelialization of wounds treated with Oleogel‐S10.

## 1 OLEOGEL‐S10: PHYSICAL AND CHEMICAL PROPERTIES

Oleogel‐S10 is a wound‐healing gel containing dry extract from *Betulae* cortex (birch bark)—also referred to as triterpene extract (Laszczyk, Jager, Simon‐Haarhaus, Scheffler, & Schempp, 2006). The gel contains 90% wt/wt sunflower oil, and 10% wt/wt dry extract from birch bark, of which the majority is betulin (72–88% wt/wt). Additional active marker substances include betulinic acid, lupeol, oleanolic acid, and erythrodiol (molecular structures are shown in Figure S1) (Bickel, 2005; European Medicines Agency, 2015). The dry extract from birch bark has galenic properties causing oils to gel, forming semisolid, viscoelastic gels with thixotropic properties. These have greater viscosity at higher temperatures than at room temperature. This thixotropic property means formulations of dry extract from birch bark liquefy under movement for conveniently application, and then revert back to a gel‐like state (Bickel, 2005; Grysko & Daniels, 2013).

## 2 STUDIES ON THE EFFECT MECHANISM OF DRY EXTRACT FROM BIRCH BARK AND ITS COMPONENTS

**Figure 1:**
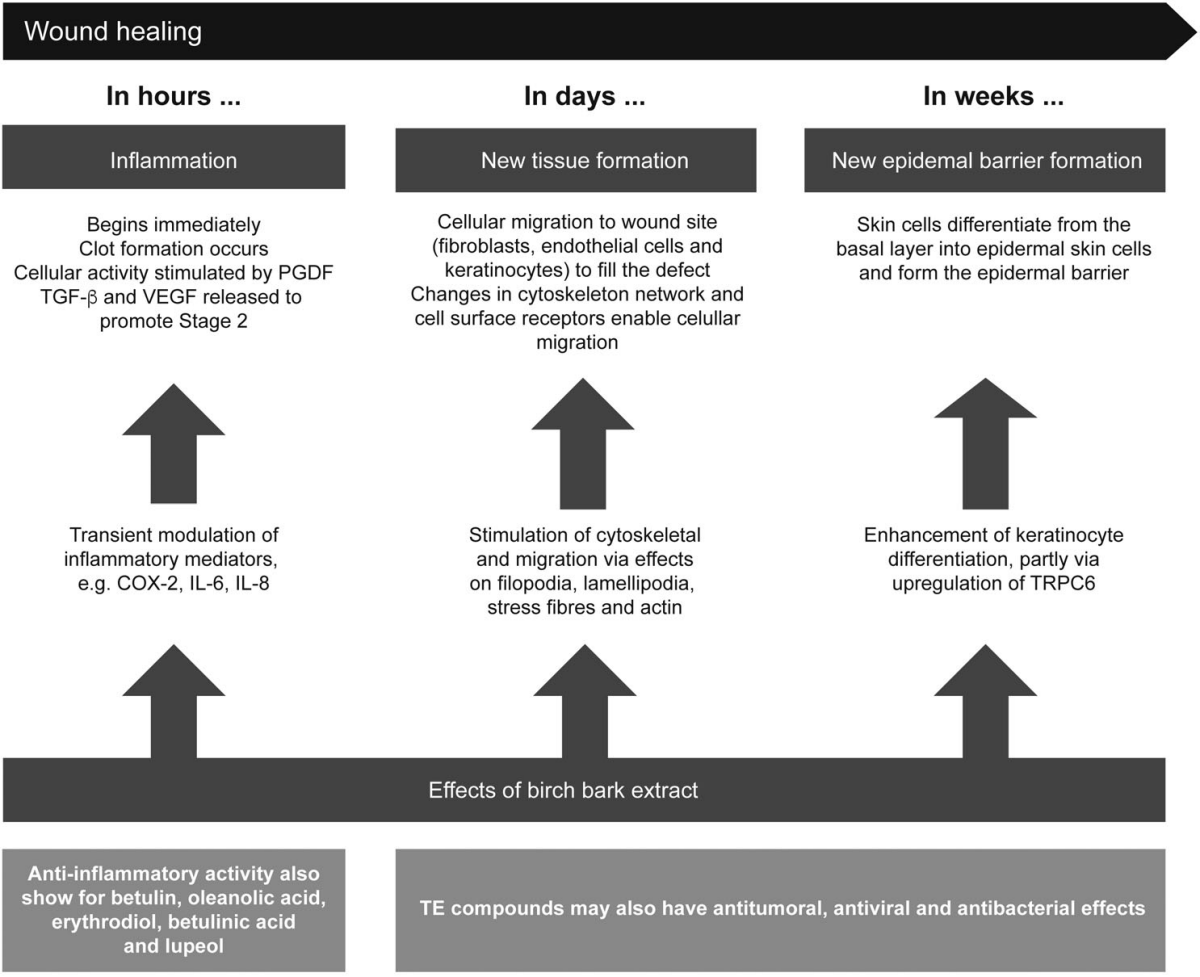
Overview of actions of dry extract from birch bark and its components during wound healing process (Stage 1, inflammation; Stage 2, tissue and epidermal barrier formation; Alakurtti et al., 2006, Ebeling et al., 2014, Laszczyk, 2009, Pastar et al., 2014, Woelfle et al., 2010). Upper panels show stages of wound healing. Lower panels show the proposed effects of dry extract from birch bark on these and other processes. Note that effects of dry extract from birch bark are determined chiefly from in vitro experiments. COX‐2, cyclooxygenase‐2; IL, interleukin; PDGF, platelet‐derived growth factor; TE, dry extract from birch bark (triterpene extract); TGF‐β, transforming growth factor‐β; TRPC6, transient receptor potential canonical (subtype) 6; VEGF, vascular endothelial growth factor

The physiological process of wound healing has been described as a three‐stage process of inflammation, tissue formation, and wound closure/remodeling (Valero, Javierre, Garcia‐Aznar, Menzel, & Gomez‐Benito, 2015). In various studies, dry extract from birch bark and its active marker substances have been shown to have activity in the first two stages of the wound healing process (Alakurtti, Makela, Koskimies, & Yli‐Kauhaluoma, 2006; Bernard, Scior, Didier, Hibert, & Berthon, 2001; Ci et al., 2017; Doller et al., 2008; Ebeling et al., 2014; Galgon, Wohlrab, & Drager, 2005; Laszczyk, 2009; Lee, Nam, Kim, & Lee, 2006; Recio, Giner, Manez, & Rios, 1995; Saleem, Afaq, Adhami, & Mukhtar, 2004; Suksamrarn et al., 2006; Takada & Aggarwal, 2003; Tseng & Liu, 2004; Wardecki et al., 2016; Woelfle et al., 2010; Yadav, Prasad, Sung, Kannappan, & Aggarwal, 2010; Yogeeswari & Sriram, 2005; Yun et al., 2003). An overview of this activity is presented in Figure 1. In the sections that follow, the effects of dry extract from birch bark are described according to these two stages.

### 2.1 Effects on inflammation

Data on the early inflammatory stages of wound healing show that dry extract from birch bark and betulin have a modulatory role where by some pro‐inflammatory mediators are upregulated and others are downregulated (Ebeling et al., 2014).

Specifically, dry extract from birch bark and betulin both upregulate pro‐inflammatory cytokines cyclooxygenase 2 (COX‐2), interleukin (IL)‐6, and IL‐8 in primary human keratinocytes cells. This upregulation occurs at the level of both RNA (within 8–24 hr) and protein (by 24 hr) (Ebeling et al., 2014). The increase in mRNA halflife of immunocompetent peptides is due to an mRNA‐stabilizing effect of dry extract from birch bark and betulin. This process involves p38 mitogen‐activated protein kinase and human antigen R (Doller et al., 2008; Ebeling et al., 2014). Additionally, increased expression of IL‐6 and COX‐2 has been confirmed in a porcine wound healing model and in human skin punch biopsies challenged with dry extract from birch bark and betulin (Ebeling et al., 2014). The increase in pro‐inflammatory cytokine expression seen in studies of dry extract of birch bark and its active marker substances are temporary and are attenuated during the course of long‐term treatment (Ebeling et al., 2014).

Anti‐inflammatory activity of betulin, oleanolic acid, erythrodiol, betulinic acid, and lupeol has been shown in various experimental models (Bernard et al., 2001; Ci et al., 2017; Laszczyk, 2009; Saleem et al., 2004; Takada & Aggarwal, 2003; Tseng & Liu, 2004; Yadav et al., 2010; Yogeeswari & Sriram, 2005). Exact mechanisms of these activities are not yet completely understood, however, and further investigations are required to fully elucidate and delineate pro‐inflammatory and anti‐inflammatory effects of Oleogel‐S10 and its various active marker substances.

### 2.2 Effects on new tissue formation

Dry extract from birch bark and its components betulin, lupeol, and erythrodiol influence the new tissue formation stage (Stage 2) of wound healing by promoting keratinocyte migration, possibly by increasing the formation of actin filopodia, lamellipodia, and stress fibers (Ebeling et al., 2014; Wardecki et al., 2016). Dry extract from birch bark and its active marker substances strongly influence the actin cytoskeleton, resulting in polarization of the cells, formation of lamellipodia and filopodia at the leading edge, and stress fibers in the cell body (Ebeling et al., 2014). Changes in actin cytoskeleton are also evident in fibroblasts exposed to triterpene extracts and betulin (Wardecki et al., 2016). Activity of dry extract from birch bark on the actin cytoskeleton is dependent on the activation of Rho GTPases (Ebeling et al., 2014).

### 2.3 Effects on forming a new epidermal barrier

Differentiation of keratinocytes is—part of the second stage of wound healing—is essential for reepithelialization and maintenance of the skin barrier. Dry extract from birch bark promotes keratinocyte differentiation in vitro and in vivo (Woelfle et al., 2010). Dry extract from birch bark stimulated enhancement of differentiation appears to be mediated at least in part by upregulation of the nonselective transient receptor potential canonical (subtype) 6 (TRPC6), which regulates keratinocyte calcium influx (Woelfle et al., 2010).

## 3 OLEOGEL‐S10: PRECLINICAL AND CLINICAL STUDIES

Processes of wound healing in epidermolysis bullosa (EB) are not fully understood (Bruckner‐Tuderman & Mellerio, 2017), and the range of mutations and different depths of cleavage define the variety of clinical phenotypes (Fine et al., 2014). Oleogel‐S10 does not treat the underlying genetic defect in EB—in vitro data have shown that the agent acts on common and general mechanisms of wound healing (Ebeling et al., 2014). These data suggest that the effects of Oleogel‐S10 on wound healing in EB would be independent of disease subtype. Both the European Medicines Agency (EMA) in 2011, and the Food and Drug Administration (FDA) in 2014, issued an orphan drug designation for the active substance betulin for the indication of EB.

Preclinical studies of Oleogel‐S10 have been conducted to evaluate safety, tolerability, and toxicity. Parenteral repeated‐dose toxicity studies in rats and dogs showed no systemic changes related to the test substance, and a pronounced inflammatory, granulomatous reaction observed at the injection site following subcutaneous administration in dogs was considered as due to the insolubility of the triterpenes (Alakurtti et al., 2006). Moreover, dermal application of Oleogel‐S10 to intact or abraded mini‐pig skin did not result in any systemic toxicity and was generally well tolerated. A high degree of tolerability was also observed for maximal systemic exposure in juvenile animals (European Medicines Agency, 2015).

**Figure 2:**
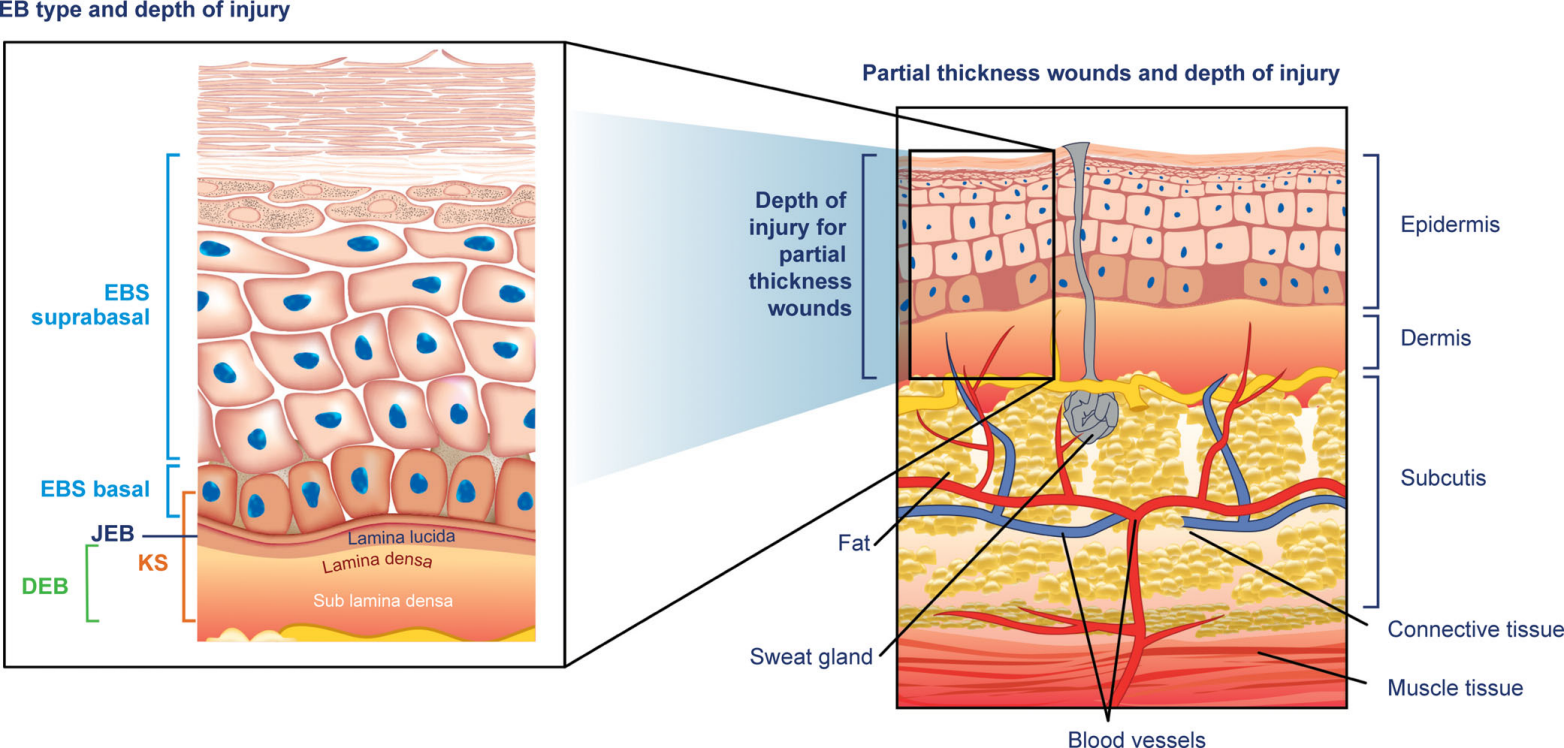
Human skin and depth of cutaneous wounds in epidermolysis bullosa. Diagram shows the relationship between depth of injury in partial thickness wounds (i.e., those sustained in graft surgery) and EB. EB, epidermolysis bullosa; EBS, EB simplex; JEB, junctional EB; KS, Kindler syndrome; DEB, dystrophic EB

Wounds of the skin can be compared to full or partial thickness wounds, based on the depth of skin layers involved. The level of skin cleavage in the major EB types/subtypes extends at maximum into the upper part of the dermis, thus EB wounds resemble partial thickness wounds (Denyer, Pillay, & Chlapham, 2017; Fine et al., 2014). Sensory nerve endings in these wounds are abundant in the remaining dermal tissue of the wound bed and there is also an increased risk of infection due to the compromised skin barrier. Human skin and depth of cutaneous wounds in EB are illustrated in Figure 2 (Fine et al., 2014; Vogt & Ipaktchi, 2013).

Clinical data are available from one Phase 2 and three Phase 3 studies of the use of Oleogel‐S10 in the treatment of partial thickness wounds of different etiologies. In the Phase 3 setting, Oleogel‐S10 was found to accelerate wound healing in partial thickness wounds in split thickness skin graft (STSG) donor site wounds (studies BSH‐12 and BSG‐12) (Barret et al., 2017), and in Grade 2a burn wounds (BBW‐11) (Frew et al., 2018). In STSG donor site wounds, the 17‐day healing period with standard treatment was reduced by 1–2 days (Barret et al., 2017). In Grade 2a burn wounds, the 9‐day healing period with standard treatment was reduced by 1 day (Frew et al., 2018). Oleogel‐S10 had a good overall safety profile in Phase 3 studies and appeared to be well tolerated. A further, single‐centre, Phase 3 study of 32 undergoing graft surgery showed quicker wound closure and better viscoelastic properties of wounds treated with Oleogel‐S10 compared with controls (Lipovy et al., 2018).

As a result of formal Phases 2 and 3 studies, Oleogel‐S10 received a marketing authorization in the European Economic Area under the tradename Episalvan® in January 2016 for the treatment of partial thickness wounds in adults. It should be noted that the safety and efficacy of Oleogel‐S10 in children and adolescents under 18 years have not yet been established.

A proof of concept Phase 2 study in patients with dystrophic EB has suggested the potential for faster reepithelialization of wounds treated with Oleogel‐S10 (Schwieger‐Briel, Kiritsi, Schempp, Has, & Schumann, 2017). The primary efficacy variable in this study was faster reepithelialization from baseline to either Day 14 or Day 28 (in case of delayed wound healing), as evaluated by two blinded experts. A secondary outcome variable was percentage of wound epithelialization. Twelve wound pairs were evaluated, in 10 patients with dystrophic EB. For each wound pair, one was treated with Oleogel‐S10 plus nonadhesive wound dressing and the other with nonadhesive wound dressing only (control). Oleogel‐S10 plus nonadhesive wound dressing accelerated the reepithelialization in eight of eight decided cases (*p* = .0078, binomial test) (Schwieger‐Briel et al., 2017). In five cases, both blinded reviewers considered epithelialization of the wounds treated with Oleogel‐S10 as superior; in three cases only one reviewer considered Oleogel‐S10 as superior and the other one as equal to control; no case was scored in favor of control. Four cases were undecided (i.e., investigators could not be decided which wound or wound half showed faster reepithelization than the other). Measurements of wound size showed a trend toward accelerated wound healing with Oleogel‐S10 but this was not statistically significant. Touch sensitivity, itching, exudation, and efficacy as rated by investigators and patients were comparable for both treatments. These results indicate a potential for faster reepithelialization of wounds in patients with dystrophic EB when treated with Oleogel‐S10 but larger studies are needed to confirm the significance of these findings (Schwieger‐Briel et al., 2017).

A global Phase 3 trial of Oleogel‐S10 in EB is currently ongoing. Efficacy And Safety of Oleogel S10 in patients with EB (EASE) (NCT03068780; EudraCT No. 2016–002066‐32) is a double‐blind, randomized, placebo‐controlled, Phase 3, efficacy and safety study with 24‐month open‐label follow‐up of Oleogel‐S10 in patients with inherited EB (EU Clinical Trials Register, 2018). The primary objective of the double‐blind phase of the trial is to compare the efficacy of Oleogel‐S10 with placebo gel in the promotion of healing of EB partial thickness wounds. This will be assessed as the incidence of the first complete closure of the EB target wound: the primary efficacy endpoint is complete closure of the EB target wound within 45 ± 7 days of treatment, and secondary objectives will include efficacy of treatment as evidenced by other criteria described in the study protocol, safety, tolerability, and exposure to betulin.

Bland creams or ointments are routinely applied to wounds to help prevent wound crusting from serous exudate. As a gel, Oleogel‐S10 may also contribute to this type of effect. Oleogel‐S10 is also sterile and its gel‐like nature is likely to act as a physical barrier to infection, which is an important consideration in the management of wounds associated with EB. In common with other patients with chronic wounds, when treating patients with significant EB‐related wound burden, possible issues with contact allergies (e.g., hydrocolloid dressings) may also need to be considered (Muller & Kiritsi, 2017). To our knowledge, there is no current literature on potential contact allergy to the individual components of Oleogel‐S10. However, there is a published case report of possible contact allergy with a cosmetically used emulsion that is based on the dry extract of birch bark used in Oleogel‐S10 (Meyer‐Hoffert & Brasch, 2013). Oleogel‐S10 is considered safe to use for people who are allergic to birch pollen, as these allergens are not present in the formulated product (Amryt, 2015).

## 4 OLEOGEL‐S10 IN CONTEXT

EB affects approximately 1 in 17,000 live births and it is estimated that there are around 500,000 people living with the condition worldwide (figures from DEBRA International). EB is caused by more than 1,500 known mutations in at least 20 genes encoding anchoring and other proteins of the dermoepidermal junction (Uitto, Bruckner‐Tuderman, McGrath, Riedl, & Robinson, 2018). More than 30 different subtypes, inherited in either an autosomal dominant or recessive manner and with varying clinical presentations, are currently recognized (Fine, 2010; Fine et al., 2014; Uitto et al., 2018).

Currently, there is no cure for EB although research is progressing, and, recently, the regeneration of a large part of the affected epidermis using transgenic stem cells was reported in a 7‐year‐old child with a life‐threatening form of junctional EB (Hirsch et al., 2017). Treatment of patients with EB consists primarily of preventive and/or palliative measures, together with supportive care to relieve itch and pain (DEBRA International, 2018; Denyer et al., 2017; El Hachem et al., 2014). Data from patients with partial thickness wounds and EB treated with Oleogel‐S10 have been encouraging, and it is possible that future clinical practice guidelines could include the agent as a component of the standard of care for EB.

## 5 CONCLUSIONS

There are many different EB genotypes and phenotypes and currently there is no effective pharmacological or genetic treatment option for all subtypes (DEBRA International, 2016). Most commentators acknowledge that causal treatment of EB will be achievable through genetic intervention, but that this is a long‐term aim due in part to the number of mutated genes involved. Moreover, considering the involved surfaces affected by fragility, even if genetic treatments become more readily available, this will not necessarily mean a complete cure of the disease. Until then, treatments to accelerate wound healing and provide relief of pain and itch are greatly appreciated and urgently needed.

Oleogel‐S10 has been shown to accelerate the reepithelialization of wounds, and this is thought to be due to a bimodal pro‐ and anti‐inflammatory effect as well as an enhancement of keratinocyte migration and differentiation. Substantiation of the results of the concept Phase 2 EB study with a randomized, placebo‐controlled Phase 3 study was started in April 2017 (EASE; NCT03068780) after a consultation with the FDA and EMA. Results are expected in 2019.

## DISCLOSURES

A.S.B., H.O., D.K., and C.B. confirm that their contribution to the manuscript is entirely independent of economic, research, or other relations to the sponsor. M.L.L. is an employee of Amryt AG, part of the Amryt Pharmaceuticals DAC group.

## Supporting information


**Figure S1** The molecular structure of the five active marker substances of the dry extract from Birch bark. **A** Lupane structure: Lupeol (R1‐OH,R2‐CH_3_); Betulin (R1‐OH, R2‐CH_2_OH); Betulinic acid (R1‐OH, R2‐COOH); **B** Oleanane structure: Erythrodiol (R1‐OH, R2‐CH_2_OH); Oleanolic acid (R1‐OH,R2‐COOH)
